# Nomogram for predicting the overall survival of underweight patients with colorectal cancer: a clinical study

**DOI:** 10.1186/s12876-023-02669-8

**Published:** 2023-02-13

**Authors:** Jun Woo Bong, Younghyun Na, Yeonuk Ju, Chinock Cheong, Sanghee Kang, Sun Il Lee, Byung Wook Min

**Affiliations:** Department of Surgery, Korea University Guro Hospital, Korea University College of Medicine, 148, Gurodong-Ro, Guro-Gu, Seoul, 08308 Republic of Korea

**Keywords:** Colorectal cancer, Underweight, Overall survival, Nomogram, Body mass index

## Abstract

**Background:**

An underweight individual is defined as one whose Body Mass Index (BMI) is < 18.5 kg/m^2^. Currently, the prognosis in patients with colorectal cancer (CRC) who are also underweight is unclear.

**Methods:**

Information on South Korean patients who underwent curative resection for CRC without distant metastasis was collected from health insurance registry data between January 2014 and December 2016. We compared the overall survival (OS) of underweight and non-underweight (BMI ≥ 18.5 kg/m^2^) patients after adjusting for confounders using propensity score matching. A nomogram to predict OS in the underweight group was constructed using the significant risk factors identified in multivariate analysis. The predictive and discriminative capabilities of the nomogram for predicting 3- and 5-year OS in the underweight group were validated and compared with those of the tumor, node, and metastasis (TNM) staging system in the training and validation sets.

**Results:**

A total of 23,803 (93.6%) and 1,644 (6.4%) patients were assigned to the non-underweight and underweight groups, respectively. OS was significantly worse in the underweight group than in the non-underweight group for each pathological stage (non-underweight vs. underweight: stage I, 90.1% vs. 77.1%; stage IIA, 85.3% vs. 67.3%; stage IIB/C, 74.9% vs. 52.1%; and stage III, 73.2% vs. 59.4%, *P* < 0.001). The calibration plots demonstrated that the nomogram exhibited satisfactory consistency with the actual results. The concordance index (C-index) and area under the receiver operating characteristic curve (AUC) of the nomogram exhibited better discriminatory capability than those of the TNM staging system (C-index, nomogram versus TNM staging system: training set, 0.713 versus 0.564, *P* < 0.001; validation set, 0.691 versus 0.548, *P* < 0.001; AUC for 3- and 5- year OS, nomogram versus TNM staging system: training set, 0.748 and 0.741 versus 0.610 and 0.601; validation set, 0.715 and 0.753 versus 0.586 and 0.579, respectively).

**Conclusions:**

Underweight patients had worse OS than non-underweight patients for all stages of CRC. Our nomogram can guide prognostic predictions and the treatment plan for underweight patients with CRC.

**Supplementary Information:**

The online version contains supplementary material available at 10.1186/s12876-023-02669-8.

## Background

Colorectal cancer (CRC) is one of the most common cancers prevalent globally, and its curative rate can be increased by 68–78% through complete resection and adjuvant chemotherapy [1, 2]. The tumor, node, and metastasis (TNM) classification of the American Joint Committee on Cancer (AJCC) has been used to predict the prognosis of patients with CRC. However, the TNM staging system is insufficient to accurately predict the prognosis in certain specific CRC groups, including older patients, those with mucinous-cell type CRC, and those with distant metastasis, because the system does not reflect the detailed characteristics of such specific groups [3–5].

According to the Asia–Pacific standards of the World Health Organization (WHO), body mass index (BMI) is categorized into three groups—underweight, < 18.5 kg/m^2^; normal, 18.5–25 kg/m^2^; and obese, > 25 kg/m^2^ [6]. Previous studies have also reported the prognosis in underweight individuals with CRC; however, most studies lacked large sample sizes because of the low proportion of underweight patients [7, 8]. Therefore, we used data from the national registry to investigate the prognosis in underweight patients and developed a nomogram to predict overall survival (OS) in underweight patients. We validated the predictive capability of the nomogram and compared it to that of the TNM staging system.

## Methods

### Patient data

In South Korea, the National Quality Assessment Program (NQAP) has been conducted since 2011 to evaluate the quality of treatment and healthcare expenditures for malignant diseases. The database is managed by the Health Insurance Review and Assessment Service (HIRA) [9]. We collected information from this database on patients who underwent curative surgery for CRC without distant metastases between 2014 and 2016. Information on patient characteristics, including age, sex, body mass index (BMI) at diagnosis, American Society of Anesthesiologists (ASA) classification, primary tumor site, pathological stage, surgical margin, cell type, adjuvant chemotherapy, number of harvested lymph nodes, and emergency operation were collected. The pathological stages were graded as stages I, IIA, IIB/C, and III according to the 7th edition of the AJCC on Cancer guidelines. The tumors were categorized according to their histological type into adenocarcinoma, mucinous adenocarcinoma, signet-ring cell carcinoma (SRCC), and others. All patients were categorized into two groups according to the cut-off value for being underweight: underweight (BMI < 18.5 kg/m^2^) and non-underweight (BMI ≥ 18.5 kg/m^2^). Patients with other malignant diseases, those undergoing neoadjuvant chemotherapy, those with < 30 days of follow-up, and those with insufficient information were excluded from the study.

### Analysis of overall survival

The OS of the two groups was analyzed using the Kaplan–Meier method and the log-rank test. In order to adjust the characteristic differences between the groups, propensity scores were generated using age, sex, ASA classification, primary tumor site, emergency operation, pathological stage, surgical margin, cell type, number of harvested lymph nodes, and adjuvant chemotherapy using the MatchIt R package.

### Statistical analysis

#### Nomogram construction

The underweight group was randomly allocated to training and validation sets. Multivariate forward stepwise Cox proportional hazards analysis was performed using variables with *P* < 0.05 in univariate analysis. A nomogram for predicting 3- and 5-year OS was constructed using the RMS package in R, with significant variables identified in the multivariate analysis.

#### Nomogram validation

The variance between the predicted and actual OS was graphically depicted using calibration plots. The concordance index (C-index) was calculated to measure the discrimination between predicted and actual OS. The area under the receiver operating characteristic curve (AUC) analysis was performed to determine the sensitivity and specificity of the nomogram. The predictive performance of the nomogram was assessed using the C-index and the AUC values were assessed using the DeLong method using rcorrp.cens in the Hmisc R package. A bootstrapping resampling approach was applied to obtain comparative bias-corrected estimates.

All discrete values, shown as frequencies or proportions, were compared using the chi-square test. All data were analyzed using SAS Enterprise Guide version 6.1 (SAS Institute, Cary, NC, USA) and R software (version 3.5.1; R Foundation for Statistical Computing, Vienna, Austria). Two-sided *P*-values < 0.05 were considered statistically significant.

## Results

### Clinical characteristics of patients

**Fig. 1:**
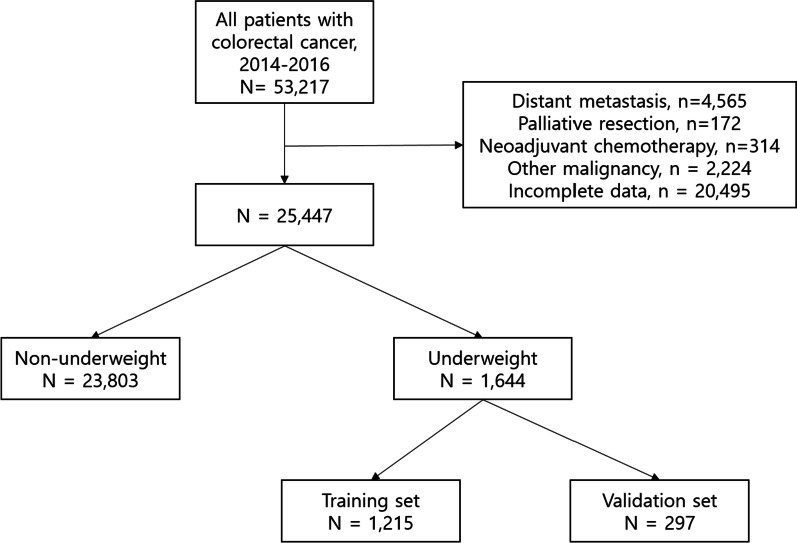
Flowchart of patient selection (non-underweight, BMI ≥ 18.5 kg/m^2^; underweight, BMI < 18.5 kg/m^2^). BMI, body mass index

From the database, 53217 patients who had undergone surgery for CRC were identified (Fig. 1). Patients who underwent palliative resection (*n* = 172), or neoadjuvant chemotherapy (*n* = 314), those with distant metastasis (*n* = 4565) or other malignancies (*n* = 2224), or who had incomplete data (*n* = 20495) were excluded. Ultimately, 25447 patients were included in the analysis. A total of 1644 (6.5%) patients were categorized into the underweight group and 23803 (93.5%) were classified into the non-underweight group. Patient characteristics are described in Table 1. The proportion of older patients, female patients, patients with worse ASA classification and advanced cancer stage, and those who underwent emergency surgery and adjuvant chemotherapy, was higher in the underweight group than in the non-underweight group.

**Table 1:** Patient clinicopathologic characteristics according to non-underweight and underweight status

	Non-underweight		Underweight		*P* value
	*N* = 23,803	%	*N* = 1,644	%	
*Age, years*					< 0.001
< 65	10,139	42.6	524	31.9	
65–75	8,147	34.2	443	26.9	
> 75	5,517	23.2	677	41.2	
Male sex	14,302	60.1	892	54.3	< 0.001
*ASA classification*					< 0.001
I–II	19,546	82.1	1,185	72.1	
III	4,015	16.9	428	26.0	
IV–VI	235	1.0	30	1.8	
*Primary tumor site*					0.622
Colon	15,570	65.4	1,065	64.8	
Rectum	8,233	34.6	579	35.2	
*Pathologic stage*					< 0.001
I	6,323	26.6	236	14.4	
IIA	7,200	30.2	578	35.2	
IIBC	1,021	4.3	127	7.7	
III	9,258	38.9	702	42.7	
Surgical margin, positive	257	1.2	25	1.6	0.187
*Cell type*					0.083
AC	21,050	95.8	1,499	94.5	
MAC	673	3.1	66	4.2	
SRCC	50	0.2	5	0.3	
Others	208	0.9	17	1.1	
Adjuvant chemotherapy, yes	10,697	47.6	657	41.8	< 0.001
*Number of harvested lymph nodes*					0.482
≥ 12	18,487	94.7	1,325	95.2	
< 12	1,032	5.3	67	4.8	
Emergency operation, yes	1,319	5.5	209	12.7	< 0.001

Underweight, BMI < 18.5 kg/m^2^; non-underweight, BMI ≥ 18.5 kg/m.^2^

*AC* adenocarcinoma; *ASA* American Society of Anesthesiologists; *MAC* mucinous adenocarcinoma; *SRCC* signet-ring cell carcinoma

### Survival analysis

**Fig. 2:**
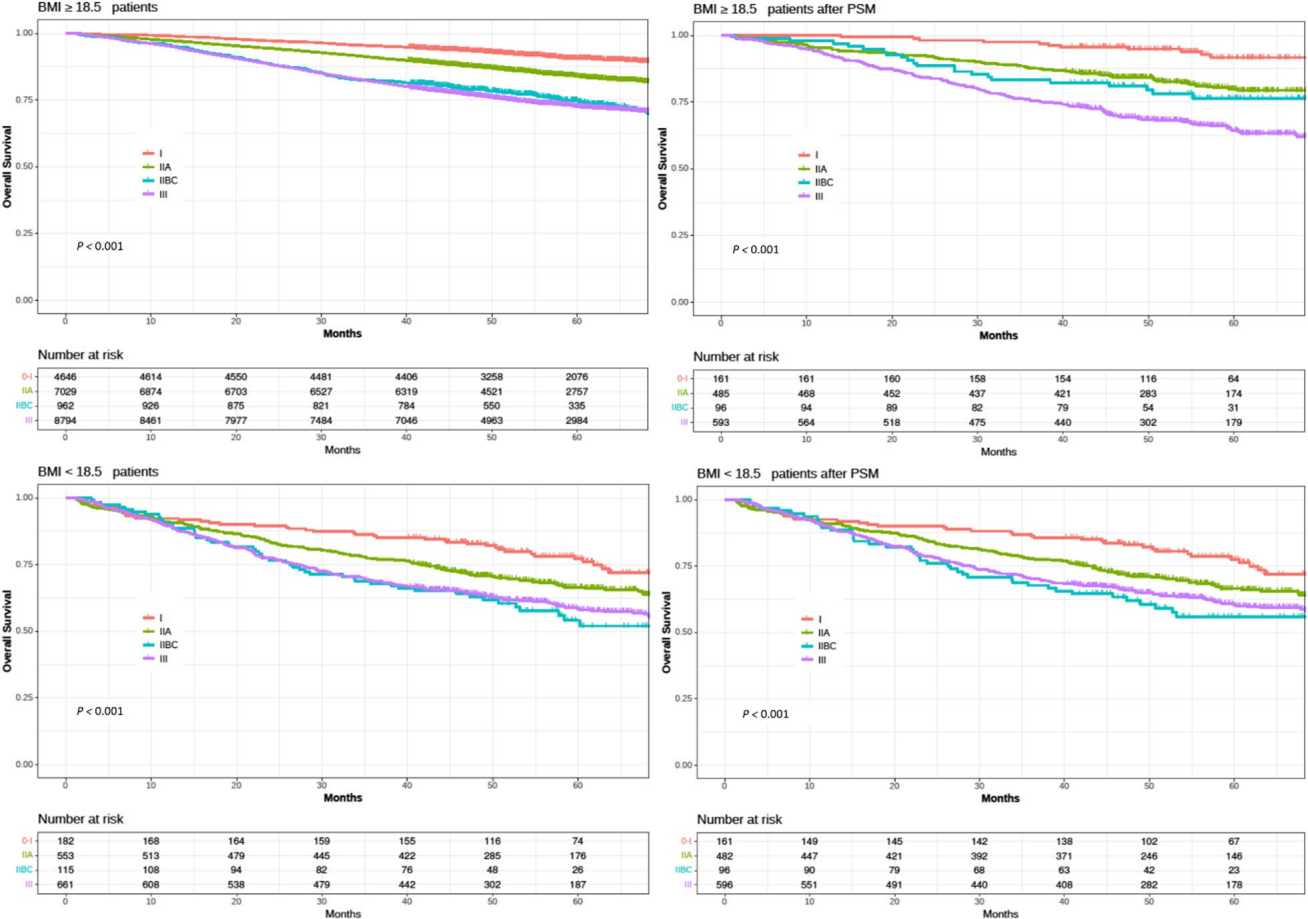
Kaplan–Meier curve for overall survival in non-underweight and underweight patients according to pathological stages I, IIA, IIB/C, and III (non-underweight, BMI ≥ 18.5 kg/m^2^; underweight, BMI < 18.5 kg/m^2^). BMI, body mass index; PSM, propensity score matching

The median follow-up period for all the patients was 52.5 months. Figure 2 shows the OS of the non-underweight and underweight groups according to the pathological stage. The 5-year OS rates for stage I, IIA, IIB/C, and III cancers in the non-underweight group were 90.1%, 85.3%, 74.9%, and 73.2%, respectively (*P* < 0.001). In the underweight group, the 5-year OS rates for stage I, IIA, IIB/C, and III cancers were 77.1%, 67.3%, 52.1%, and 59.4%, respectively (*P* < 0.001). After propensity score matching, 1,335 patients were allocated to each group (Additional File 1). In the non-underweight group, the 5-year OS rates for stage I, IIA, IIB/C, and III cancers were 90.2%, 80.8%, 76.2%, and 63.1%, respectively (*P* < 0.001). In the underweight group, the 5-year OS rates for stages I, IIA, IIB/C, and III cancers were 76.2%, 66.7%, 56.2%, and 60.2%, respectively (*P* < 0.001). The OS of the underweight group was worse than that of the non-underweight group for all stages.

### Nomogram development

**Fig. 3:**
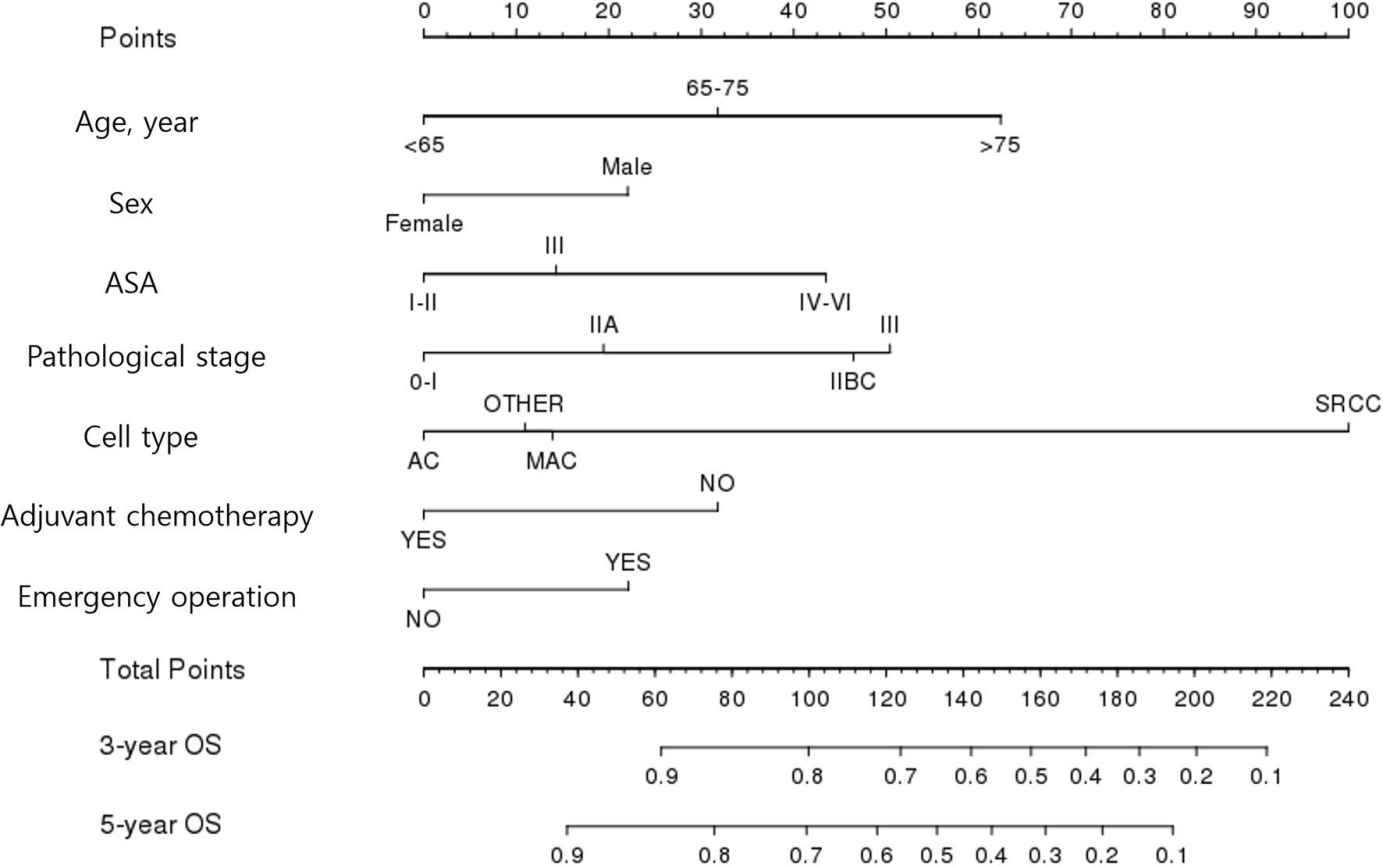
Nomogram for overall survival in underweight patients with BMI < 18.5 kg/m^2^ BMI, body mass index; ASA, American Society of Anesthesiologists; AC, adenocarcinoma; MAC, mucinous adenocarcinoma; SRCC, signet-ring cell carcinoma; OS, overall survival

A total of 1,215 and 297 patients were randomly allocated to the training and validation sets, respectively, in an 8:2 ratio (Additional File 2). In the training set, univariate analysis revealed that eight variables could affect OS (Table 2). Among them, seven variables, including age, sex, ASA classification, pathological stage, cell type, emergency operation, and adjuvant chemotherapy, were significantly associated with OS according to multivariate analysis (*P* < 0.05), and a nomogram for predicting 3- and 5-year OS was established (Fig. 3). Cell type was the most significant factor for OS, followed by age, pathological stage, ASA classification, adjuvant chemotherapy, emergency operation, and sex.

**Table 2:** Univariate and multivariate analyses of the risk factors for overall survival in underweight patients

			Univariate analysis				Multivariate analysis		
			HR	95% CI	*P* value		HR	95% CI	*P* value
Age, years	< 65		1				1		
	65–75		1.91	1.41–2.58	< 0.001		1.86	1.34–2.59	< 0.001
	> 75		3.84	2.95–5.01	< 0.001		3.40	2.50–4.61	< 0.001
Sex	Male		1				1		
	Female		0.83	0.69–1.00	0.045		0.64	0.52–0.79	< 0.001
ASA classification	I, II		1				1		
	III		1.79	1.47–2.19	< 0.001		1.32	1.07–1.63	< 0.001
	IV–VI		2.55	1.39–4.79	0.004		2.34	1.19–4.62	0.013
Primary tumor site	Colon		1				1		
	Rectum		1.29	1.07–1.57	0.007		1.08	0.84–1.23	0.124
Pathological stage	I		1				1		
	IIA		1.47	1.01–2.12	0.043		1.46	0.99–2.14	0.051
	IIB/C		2.32	1.48–3.63	< 0.001		2.48	1.55–3.98	< 0.001
	III		2.06	1.44–2.94	< 0.001		2.68	1.83–3.94	< 0.001
Cell type	AC		1				1		
	MAC		1.80	1.25–2.58	0.001		1.31	0.90–1.91	0.154
	SRCC		2.04	0.51–8.18	0.315		6.92	1.68–28.50	0.007
	Others		1.03	0.42–2.49	0.943		1.23	0.50–3.01	0.636
Adjuvant chemotherapy	No		1.96	1.59–2.40	< 0.001		1.87	1.48–2.35	< 0.001
Number of harvested lymph nodes	≥ 12		1						
	< 12		1.42	0.91–2.20	0.12				
Emergency operation		Yes	1.77	1.37–2.28	< 0.001		1.54	1.18–2.01	0.001

*ASA* American Society of Anesthesiologists; *AC* adenocarcinoma; *MAC* mucinous adenocarcinoma; *SRCC* signet-ring cell carcinoma

### Nomogram validation

**Fig. 4:**
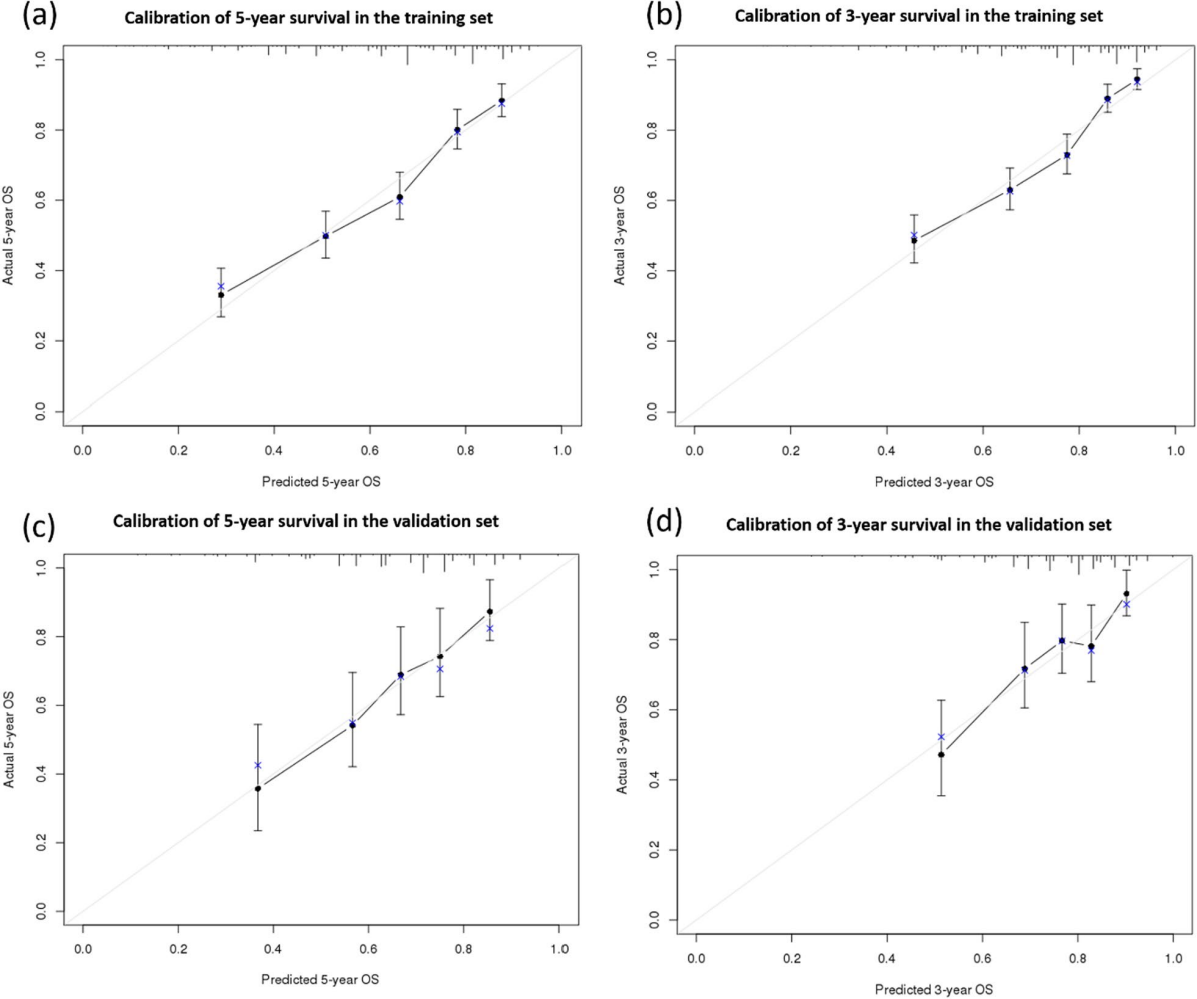
Calibration plot for **a** 5-year overall survival in the training set, **b** 3-year overall survival in the training set, **c** 5-year overall survival in the validation set, and **d** 3-year overall survival in the validation set

**Fig. 5:**
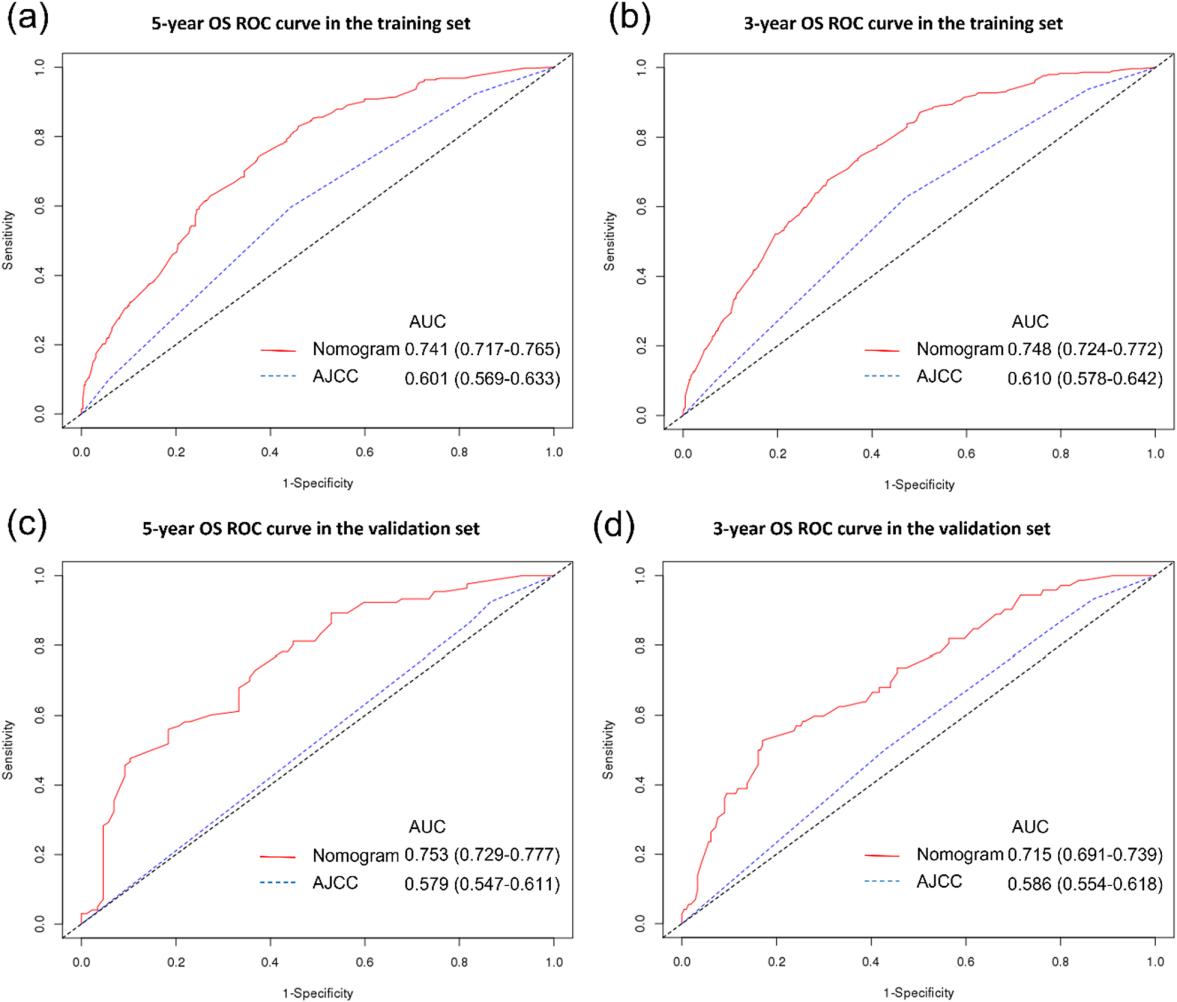
Discriminatory accuracy according to receiver operating characteristics analysis for predicting overall survival (OS); 5-year **a** and 3-year **b** OS in the training set; 5-year **c** and 3-year **d** OS in the validation set (red line, nomogram-predicted curve; blue dotted line, TNM staging system-predicted curve) ROC, receiver operating characteristic; AUC, area under ROC curve

The calibration plots indicate a high correlation between the predicted and actual results in the training and validation sets (Fig. 4). In the training set, the nomogram had a superior ability to discriminate OS compared to the TNM staging system (C-index [95% confidence interval {CI}], 0.713 [0.689–0.737] *vs.* 0.564 [0.538–0.589], *P* < 0.001, Table 3). In addition, in the validation set, the discriminative capability of the nomogram was higher than that of the TNM staging system (C-index [95% CI]: 0.691 [0.638–0.744] *vs.* 0.548 [0.494–0.602], *P* < 0.001). Furthermore, in the training set, the AUC of the nomogram prediction model for predicting 3- and 5- year OS were 0.748 and 0.741, respectively, whereas those of the TNM staging system were 0.610 and 0.601, respectively (Fig. 5). In the validation set, the AUC for predicting the 3- and 5- year OS of the nomogram prediction model were 0.715 and 0.753, respectively, whereas those of the TNM staging system were 0.586 and 0.579, respectively.

**Table 3:** C-indices for the nomogram and TNM staging system in underweight patients

	Training set		Validation set	
	C-index (95% CI)	*P* value	C-index (95% CI)	*P* value
Nomogram	0.713 (0689–0.737)		0.691 (0.638–0.744)	
TNM staging system	0.564 (0.538–0.589)	< 0.001	0.548 (0.494–0.602)	< 0.001

*HR* hazard ratio; *CI* confidence interval

## Discussion

In the present study, underweight patients with CRC had significantly worse OS than non-underweight patients with CRC at each TNM stage. We developed a nomogram for predicting OS in underweight patients with CRC and validated its favorable performance. Our nomogram showed a greater predictive ability for OS than did the TNM staging system (Table 3, Fig. 5).

The relationship between BMI and the prognosis of malignant disease has been reported in pancreatic, breast, stomach, and lung cancers [10–13]. In CRC, BMI is a significant prognostic marker [14]. A positive correlation between obesity and a higher risk of CRC has been demonstrated in previous studies [15, 16]. Although obesity is associated with a higher risk of CRC, overweight and low-class obese patients show better survival than underweight, normal, and high-class obese patients [17, 18]. This phenomenon, the obesity paradox, supports the fact that extra weight is essential for better nutritional status and appropriate body composition to manage the metabolic requirements of cancer treatments [19, 20].

In most previous studies, low BMI was an independent risk factor for a worse prognosis. Among the patients with CRC, underweight patients had worse OS than non-underweight patients (relative risk, 1.63; 95% CI, 1.18–2.23; *P* < 0.01) [21]. Another study reported that underweight patients with CRC showed significantly worse 10-year OS outcomes (21%; 95% CI, 12.1–36.3) than normal (40.3%; 95% CI, 36.9–43.9) or overweight (46.2%; 95% CI, 43.2–49.3) patients [22]. Kaneko et al. showed that being underweight was a significant factor for a worse prognosis, even in patients aged > 75 years [23]. Underweight patients with metastatic CRC showed worse progression-free survival than non-underweight patients [17, 24]. In this study, the prognosis of underweight patients with CRC was worse than that of non-underweight patients with CRC at stages I, II, and III. After propensity score matching between the two groups, the results remained the same. In addition, the differences in the Kaplan–Meier curves between the various stages in the underweight group were weaker than those in the non-underweight group. Moreover, in the underweight group, the OS of the patients with stage IIB/C cancer was indistinguishable from that of the patients with stage III cancer. These results support the fact that the TNM staging system does not sufficiently represent the survival of underweight patients, and there may be limitations in predicting prognosis using the TNM staging system.

Previous studies have reported that a low BMI can hinder adherence to chemotherapy because there is a strong correlation between low BMI and chemotherapy toxicity, such as mucositis and anorexia, has been reported [25, 26]. Consequently, chemotherapy and the toxicity of chemotherapeutic agents can directly induce body weight loss [27, 28]. A low reserve of body composition may restrict the opportunity to receive appropriate postoperative therapy especially after recurrence. In addition, sarcopenia is a risk factor for poor OS in patients with CRC [29]. An imbalance in cytokines associated with sarcopenia may worsen cancer prevention [30, 31]. A positive correlation between BMI and sarcopenia has been reported previously [31, 32]. Thus, sarcopenia may be a significant factor affecting the poor prognosis of underweight patients in this study. Other studies, moreover, have suggested that tumor aggressiveness in underweight patients is worse, even in the early stages. In previous randomized trials, underweight patients with CRC showed increased tumor aggressiveness and significantly shorter recurrence-free survival than non-underweight patients in a 5-fluorouracil-based adjuvant chemotherapy setting [33]. Other studies on stage I–III CRC have reported that underweight patients (BMI < 20 kg/m^2^) showed early recurrence and poor prognosis [33, 34].

In our nomogram, SRCC was the risk factor with the greatest contribution to OS. In this study, the HR of SRCC in underweight patients was 6.92 (95% CI 1.68–28.50), which was higher than the HRs in previous reports with patients of all body weights (1.58–3.77) [35]. SRCC is defined by tumor cells in the presence of > 50% intracytoplasmic mucin and accounts for 1% of CRC cases [36]. Compared to other types of CRC, SRCC tends to have a poorer prognosis, with early onset, right-sidedness, and peritoneal metastasis [37]. Although the nature of SRCC is significantly aggressive and the prognosis or treatment of SRCC is difficult to predict, other studies have reported that surgery and adjuvant chemotherapy still have a significant role in improving the survival of patients with SRCC [4, 38]. Therefore, we need to consider active surgery and chemotherapy for CRC patients with SRCC, even those who are underweight. Age > 75 years was the second most significant factor for OS in our nomogram. Previous studies have reported that elderly patients with CRC show unique characteristics of poor cancer-specific survival and right-sidedness [39]. Other studies have demonstrated that old age is not a significant factor affecting CRC-specific death. Although the debate regarding the effect of old age on the prognosis of CRC is ongoing, most studies have reported that elderly patients showed higher trends of postoperative complications and non-administration of chemotherapy [40]. Thus, old age is another significant negative factor in the prognosis of underweight CRC patients, affecting adjuvant therapy or treatment after recurrence.

The underweight cutoff value in this study was based on the WHO obesity classification. Although it is an internationally accepted value, previous studies have reported that it is necessary to set different cut-off values for BMI as a surrogate marker to determine the prognosis of CRC [34, 41]. As the proportion of patients with obesity has been increasing in many developed nations, the cut-off value may change over time and in different populations. Further studies are required to determine the cut-off value for predicting CRC prognosis more accurately.

One of the limitations of this study is that the NQAP database of the HIRA in South Korea did not provide detailed information on variables such as T/N categories and postoperative complications. In addition, OS was the only available record pertaining to survival; thus, information on cancer-specific or recurrence-free survival was unavailable. Moreover, because the South Korean population is a single ethnic group with a relatively high population density and uniformity, the fact that external validation was conducted in the same cohort is another limitation of this study. Therefore, in order to apply the results of this study to various races and countries, additional analysis is needed using national databases in which information on BMI is available.

One of the strengths of our study is that it provides a nomogram for the OS of underweight CRC patients based on the national registry. In addition, BMI and other variables in this nomogram are simple tools that can be used in clinical practice. Furthermore, this nomogram showed superior predictive performance and reliability compared with the conventional TNM staging system. This study found that underweight patients showed poorer survival than non-underweight patients, even in the early stages, and the survival differences in OS between the different stages in underweight patients were smaller than those in non-underweight patients. Therefore, we should carefully determine postoperative treatments and follow-up periods, and pay attention to predicting the survival of underweight patients with CRC more accurately.

In conclusion, we constructed and validated a nomogram to predict the 3- and 5-year OS rates of underweight patients with CRC (BMI < 18.5 kg/m^2^). The established nomogram could be used to predict the prognosis of underweight patients with better accuracy.

## Supplementary Information

**Additional file 1**. Clinicopathological characteristics of non-underweight and underweight patients after propensity score matching.

**Additional file 2**. Clinical characteristics of underweight patients after division into training and validation sets.

## Data Availability

The data of the National Quality Assessment Program are not publicly accessible and are available only with permission from the Health Insurance Review and Assessment Service. The corresponding author will provide a permission of the access to the database on reasonable request.
