# Supplementary material for: Nomogram for predicting the overall survival of underweight patients with colorectal cancer: a clinical study
Source: BMC Gastroenterol. 2023 Feb 13;23:39. doi: 10.1186/s12876-023-02669-8 (PMC9923908; doi:10.1186/s12876-023-02669-8)
Supplement: Supplementary file 1 — Additional file 1. Clinicopathological characteristics of non-underweight and underweight patients after propensity score matching. [file 12876_2023_2669_MOESM1_ESM.docx]

**Additional file 1.** Clinicopathological characteristics of non-underweight and underweight patients after propensity score matching

|  | Non-underweight | | | | Underweight | | | | *P*-value |
| --- | --- | --- | --- | --- | --- | --- | --- | --- | --- |
|  | N=1,335 | | % | | N=1,335 | | % | |  |
| Age, years |  |  | |  | |  | | 0.974 | |
| <65 | 393 | 29.8 | | 403 | | 30.2 | |  | |
| 65–75 | 365 | 27.3 | | 365 | | 27.3 | |  | |
| >75 | 572 | 42.8 | | 567 | | 42.5 | |  | |
| Sex, male | 733 | 54.9 | | 727 | | 54.5 | | 0.846 | |
| ASA classification |  |  | |  | |  | | 0.845 | |
| I–II | 975 | 73.0 | | 978 | | 73.3 | |  | |
| III | 345 | 25.8 | | 345 | | 25.8 | |  | |
| IV–VI | 15 | 1.1 | | 12 | | 0.9 | |  | |
| Primary tumor site |  |  | |  | |  | | 0.499 | |
| Colon | 943 | 70.6 | | 926 | | 69.4 | |  | |
| Rectum | 392 | 29.4 | | 409 | | 30.6 | |  | |
| Pathological stage |  |  | |  | |  | | 0.999 | |
| I | 161 | 12.1 | | 161 | | 12.1 | |  | |
| IIA | 485 | 36.3 | | 482 | | 36.1 | |  | |
| IIBC | 96 | 7.2 | | 96 | | 7.2 | |  | |
| III | 593 | 44.4 | | 596 | | 44.6 | |  | |
| Cell type |  |  | |  | |  | | 0.706 | |
| AC | 1,276 | 95.6 | | 1,268 | | 95.0 | |  | |
| MAC | 48 | 3.6 | | 50 | | 3.7 | |  | |
| SRCC | 3 | 0.2 | | 4 | | 0.3 | |  | |
| Others | 8 | 0.6 | | 13 | | 1.0 | |  | |
| Adjuvant chemotherapy, yes | 570 | 42.7 | | 578 | | 43.3 | | 0.784 | |
| Number of harvested lymph nodes |  |  | |  | |  | | 0.924 | |
| ≥12 | 1,279 | 95.8 | | 1,277 | | 95.7 | |  | |
| <12 | 56 | 4.2 | | 58 | | 4.3 | |  | |
| Emergency operation, yes | 126 | 9.4 | | 128 | | 9.6 | | 0.947 | |

Underweight, BMI <18.5 kg/m^2^; non-underweight, BMI ≥18.5 kg/m^2^. ASA, American Society of Anesthesiologists; AC, adenocarcinoma; MAC, mucinous adenocarcinoma; SRCC, signet-ring cell carcinoma
