# Supplementary material for: Nomogram for predicting the overall survival of underweight patients with colorectal cancer: a clinical study
Source: BMC Gastroenterol. 2023 Feb 13;23:39. doi: 10.1186/s12876-023-02669-8 (PMC9923908; doi:10.1186/s12876-023-02669-8)
Supplement: Supplementary file 2 — Additional file 2. Clinical characteristics of underweight patients after division into training and validation sets. [file 12876_2023_2669_MOESM2_ESM.docx]

**Additional file 2.** Clinical characteristics of underweight patients after division into training and validation sets

|  | Training | | | | Validation | | | | *P*-value |
| --- | --- | --- | --- | --- | --- | --- | --- | --- | --- |
|  | N=1,215 | | % | | N=297 | | % | |  |
| Age, years |  |  | |  | |  | | 0.701 | |
| <65 | 387 | 31.9 | | 89 | | 30.0 | |  | |
| 65–75 | 332 | 27.3 | | 79 | | 26.6 | |  | |
| >75 | 496 | 40.8 | | 129 | | 43.4 | |  | |
| Sex, male | 667 | 54.9 | | 161 | | 54.2 | | 0. 882 | |
| ASA classification |  |  | |  | |  | | 0. 811 | |
| I–II | 893 | 73.6 | | 213 | | 71.7 | |  | |
| III | 305 | 25.1 | | 80 | | 26.9 | |  | |
| IV–VI | 16 | 1.3 | | 4 | | 1.3 | |  | |
| Primary tumor site |  |  | |  | |  | | 0.727 | |
| Colon | 797 | 65.6 | | 191 | | 64.3 | |  | |
| Rectum | 418 | 34.4 | | 106 | | 35.7 | |  | |
| Pathological stage |  |  | |  | |  | | 0.962 | |
| I | 148 | 12.2 | | 34 | | 11.5 | |  | |
| IIA | 444 | 36.5 | | 109 | | 36.8 | |  | |
| IIBC | 94 | 7.7 | | 21 | | 7.1 | |  | |
| III | 529 | 43.5 | | 132 | | 44.6 | |  | |
| Cell type |  |  | |  | |  | | 0.053 | |
| AC | 1,139 | 93.7 | | 290 | | 97.6 | |  | |
| MAC | 57 | 4.7 | | 4 | | 1.3 | |  | |
| SRCC | 4 | 0.3 | | 1 | | 0.3 | |  | |
| Others | 15 | 1.2 | | 2 | | 0.7 | |  | |
| Adjuvant chemotherapy, yes | 522 | 44.6 | | 119 | | 41.9 | | 0.447 | |
| Number of lymph nodes harvested |  |  | |  | |  | | 0.801 | |
| ≥12 | 1,029 | 95.5 | | 249 | | 96.1 | |  | |
| <12 | 48 | 4.5 | | 10 | | 3.9 | |  | |
| Emergency operation, yes | 138 | 11.4 | | 40 | | 13.5 | | 0.362 | |

Underweight, BMI <18.5 kg/m^2^; non-underweight, BMI ≥18.5 kg/m^2^. ASA, American Society of Anesthesiologists; AC, adenocarcinoma; MAC, mucinous adenocarcinoma; SRCC, signet-ring cell carcinoma
